# Supplementary material for: Colobops: a juvenile rhynchocephalian reptile (Lepidosauromorpha), not a diminutive archosauromorph with an unusually strong bite
Source: R Soc Open Sci. 2020 Mar 25;7(3):192179. doi: 10.1098/rsos.192179 (PMC7137947; doi:10.1098/rsos.192179)
Supplement: Supplementary Figure S1 and Supplementary Notes [file rsos192179supp1.pdf]

Supplementary Information for:

Royal Society Open Science

***Colobops*: a juvenile rhynchocephalian reptile (Lepidosauromorpha), not a diminutive archosauromorph with an unusually strong bite**

Torsten M. Scheyer, Stephan N. F. Spiekman, Hans-Dieter Sues, Martín D. Ezcurra, Richard J. Butler, Marc. E. H. Jones

Supplementary Figures

Supplementary Figure S1. Surface rendering of the skull of a very early juvenile *Sphenodon punctatus*. (**a**) ventral view. (**b**) dorsal view. (**c**) left lateral view. The soft-tissue of the skull is indicated by the transparent grey area.

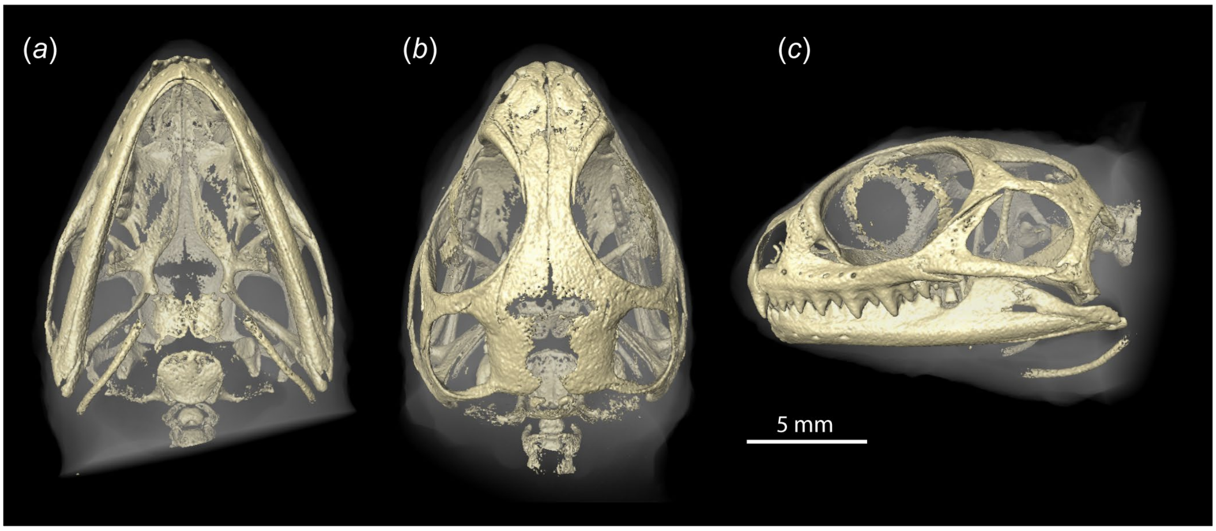

Supplementary Notes

Additional information on the performed analysis 2

Colobops noviportensis possesses the following apomorphic character-states that support its phylogenetic placement among Lepidosauromorpha and its lesser inclusive clades: frontal with transverse development posterior to the contribution to the orbital margin broader than the transverse width of the bone on the orbital margin (synapomorphy of Lepidosauromorpha); postfrontal with participation in the border of the supratemporal fenestra (synapomorphy of Rhynchocephalia), postorbital-squamosal contact continues ventrally for much or most of the ventral length of the squamosal and squamosal contacts jugal (synapomorphy of Rhynchocephalia); postfrontal with bifid medial contact, fitting broadly across both parietal and frontal (synapomorphy of Rhynchocephalia); nasal lateral surface meets entire dorsoventral height of medial surface of supra-alveolar portion of maxilla (synapomorphy of Rhynchocephalia); pterygoid with posterolaterally orientation lateral ramus, forming an obtuse angle with the anterior ramus (synapomorphy of *Planocephalosaurus* + Eusphenodontia); lacrimal absent as a separate ossification (synapomorphy of *Planocephalosaurus* + Eusphenodontia); snout with lateral margin anterior to the prefrontal formed by the nasal and maxilla with gently rounded transition along the maxilla from the lateral to dorsal side of rostrum (synapomorphy of *Clevosaurus hudsoni* + *Colobops noviportensis* + *Sphenodon punctatus*.); frontal and parietal not fused to its counterpart, respectively (synapomorphies of *Clevosaurus hudsoni* + *Colobops noviportensis* + *Sphenodon punctatus*); premaxilla with anteroventral portion developed as a ventrally oriented beak-like osseous projection (synapomorphy of *Clevosaurus hudsoni* + *Colobops noviportensis* + *Sphenodon punctatus*); prefrontal with ventral portion possessing a distinct, large notch that forms the dorsal border of a laterally opened naso-lacrimal duct (=lacrimal foramen) (synapomorphy of *Clevosaurus hudsoni* + *Colobops noviportensis* + *Sphenodon punctatus*); postfrontal equivalent in size to postorbital (synapomorphy of *Colobops noviportensis* + *Sphenodon punctatus*.); and squamosal with anterior process forming most of the lateral border of the supratemporal fenestra (synapomorphy of *Colobops noviportensis* + *Sphenodon punctatus*.).

If the poorly known *Paliguana whitei* (species represented by a poorly preserved skull) is pruned a posteriori from the analysis, the following apomorphies of Lepidosauromorpha or its lesser inclusive clades present in *Colobops noviportensis* are added: nasal total length versus frontal total length = 0.68–0.79 (synapomorphy of Lepidosauromorpha); palatine with broad transverse extension, in which the palatine is the main component of the palate posteriorly to the choanae (synapomorphy of Lepidosauromorpha); lower jaw with distinct dorsal process behind the alveolar margin formed by a dorsally well-developed posterodorsal ramus of the dentary and sometimes a dorsally well-developed coronoid bone (synapomorphy of Lepidosauromorpha); and postfrontal with medial margin dorsal relative to parietal, with extensive overlapping over the parietal (synapomorphy of Rhynchocephalia).

Characters added to the data matrix [1] as modified by subsequent authors (previous latest version published by [2]):

696. Premaxilla, anteroventral portion developed as a ventrally oriented beak-like osseous projection: absent (0); present (1).

697. Nasal, lateral surface: meets dorsoventrally short length of medial surface of dorsal process/portion of the maxilla (0); meets entire dorsoventral height of medial surface of supra-alveolar portion of maxilla (1) ([3]: character 311).

698. Lacrimal, separate ossification: present (0); absent (1) ([4]; [5]: character 27).

699. Jugal, contact with ventral process of squamosal on the posteroventral corner of the infratemporal fenestra: absent (0); present (1). This character is inapplicable in taxa lacking an infratemporal fenestra or a posterior process of the jugal and/or a ventral process of the squamosal.

700. Prefrontal, ventral portion with a distinct, large notch that forms the dorsal border of a laterally opened naso-lacrimal duct (=lacrimal foramen): absent (0); present (1).

701. Frontal, transverse development posterior to the contribution to the orbital margin: narrower than, or subequal to, the transverse width of the bone on the orbital

margin (0); broader than the transverse width of the bone on the orbital margin (1) (modified from [6]: character 15).

702. Postfrontal-parietal, contact between bones on the dorsal surface of the skull roof: present (0); absent (1). This character is inapplicable if the postfrontal bone is absent or it fused to the postorbital.

703. Postfrontals, medial margin position relative to parietal: anterior (0); lateral (1); anterior and lateral (2); dorsal, extensive overlapping over the parietal (3) ([7]; reworded from [5]: character 59 in part). This character is inapplicable in taxa lacking a postfrontal-parietal contact or a postfrontal bone.

704. Squamosal, anterior margin, bifid facet for postorbital: absent (0); present (1) ([5]: character 55).

705. Prootic, alar crest (conspicuous anterodorsal development of the prootic as a subrectangular process usually longer than the pila antotica): absent (0); present (1) ([4]; [5]: character 142).

706. Lower jaw, mandibular foramen: absent (0); present (1).

707. Splenial: present (0); absent (1) ([8]; [5]: character 176).

708. Surangular, fused to articular: absent (0); present, forming an articular complex (1) ([8]; [5]: character 188 in part).

709. Clavicle, secondary curvature anteroposteriorly: absent (0); present (1) ([8]; [5]: character 287).

710. Carpals, distal carpal 1 ossification: present (0); absent (1) ([9]; [5]: character 324).

711. Ilium, anterior pubic process: absent (0); present, a tapering process on the ilium extends over the anteroproximal surface of the pubis (1) ([8]; [6]: character 165).

Modified characters from previous version of the data matrix:

Character 5) Skull, dermal sculpturing on the dorsal surface of the skull roof: absent (0); pits or low tubercles/ridges scattered across surface (1); prominent ridges or tubercles on frontals, parietals, and nasals (2).

Character 39) Premaxilla, postnarial process: fits between the nasal and the maxilla or lies on the anterodorsal surface of the maxilla, always lateral to these bones (0); overlaps the anterodorsal surface of the nasal (1); fits into slot of the nasal (2); is overlapped by an anterolateral lamina of the maxilla (3).

Character 100) Jugal, length of the posterior process versus the height of its base: posterior process absent (0); 0.49-1.27 (1); 1.59-3.77 (2); 4.07-5.37 (3). ORDERED.

Character 187) Vomer, teeth: present, more than one row or no rows are distinguishable (0); present, mainly in a single row, but multiple teeth present immediately anterior to the contact with the pterygoid (1); present, single row along entire extension or single tooth (2); absent (3). ORDERED.

Character 207) Ectopterygoid, anteroposterior expansion of the distal end of the lateral process in dorsal or ventral view: absent or only anterior expansion present (0); anterior and posterior expansions present, fan-shaped process (1); anterior expansion absent and posterior expansion developed as a hook-shaped process (2). (Reworded from its original formulation based on [7] and [5]: character 114).

Character 351) Dorsal vertebrae, length of the centrum versus height of the centrum in anterior dorsal vertebrae: 0.45-1.10 (0); 1.18-2.00 (1); 2.19-2.90 (2). ORDERED.

Character 352) Dorsal vertebrae, length of the centrum versus height of the centrum in posterior dorsal vertebrae: 0.66-1.39 (0); 1.48-1.86 (1); 1.95-2.04 (2); 2.39-2.46 (3); >2.70 (4). ORDERED.

Character 377) Caudal vertebrae, length of the transverse process + rib versus length across zygapophyses in anterior caudal vertebrae: 0.29-0.41 (0); 0.62-1.32 (1); 1.51-1.73 (2); 2.20-2.72 (3). ORDERED.

Character 446) Metacarpus, length of the longest metacarpal versus length of the longest metatarsal: 0.34-0.45 (0); 0.51-0.59 (1); 0.74-0.98 (2). ORDERED.

Character 567) Metatarsus, length of the longest metatarsal versus length of the tibia: 0.20-0.23 (0); 0.29-0.34 (1); 0.37-0.59 (2); 0.62-0.70 (3). ORDERED.

Scoring changes from previous version of the data matrix:

Character 6: changed from (1) to (0) in *Petrolacosaurus* and *Gephyrosaurus*.

Character 36: changed from (0) to (1) in *Gephyrosaurus* and *Planocephalosaurus*.

Character 39: changed from (0) to (3) in *Mesosuchus*, *Rhynchosaurus*, *Bentonyx*, *Stenaulorhynchus*, *Brasinorhynchus*, *Langeronyx* and *Isalorhynchus*; from (0) to (?) in *Ammorhynchus*; and from (-) to (3) in *Gephyrosaurus* and *Planocephalosaurus*.

Character 95: changed from (1) to (0) in *Gephyrosaurus* and from (1) to (?) in *Planocephalosaurus*.

Character 100: changed to (0) in terminals previously scored as (-) and changed from (0) to (1), (1) to (2), and (2) to (3) in other terminals.

Character 166: changed from (1) to (0) in *Simoedosaurus*.

Character 207: terminals scored as (0) changed to (1) and those scored as (1) changed to (2), with exception of *Prolacertoides* changed from (0) to (?), *Stenaulorhynchus* and *Cuyosuchus* changed from (1) to (?), *Prolacerta* changed from (?) to (2).

Character 303: changed from (2) to (?) in *Paliguana*.

Character 307: changed from (0) to (1) in *Planocephalosaurus* based on Fraser and Walkden (1983).

Character 353: changed from (0) to (1) in *Gephyrosaurus*.

Ratios calculated for the scoring of the terminals added here:

Clevosaurus hudsoni

Character 2: 0.32 ([10]: fig. 36 and table 2).

Character 20: 0.30 ([10]: fig. 3).

Character 21: 0.51 ([10]: fig. 3).

Character 76: 0.85 ([10]: fig. 3).

Character 100: ca. 4.2 ([11]: fig. 6i).

Character 177: 112° ([10]: fig. 3).

Character 266: 0.22 ([10]: fig. 19c).

Character 331: 1.89 ([11]: fig. 15e).

Character 377: 1.32 ([10]: fig. 26a).

Character 387: 3.51 ([10]: fig. 28).

Character 416: 0.39 ([10]: fig. 29c).

Character 424: 0.47 ([10]: fig. 29c).

Character 435: 0.71–0.86 ([10]: tables 2, 3, ulna versus humerus).

Character 463: 0.84 ([10]: fig. 31).

Character 472: 1.22 ([10]: fig. 31).

Character 482: 1.42 ([10]: fig. 31).

Character 489: 1.09−1.55 ([10]: table 2).

Character 489: ca. 1.5 ([10]: fig. 4).

Character 510: 0.20 ([10]: fig. 33c).

Character 516: 0.70−0.90 ([10]: table 2).

Character 556: 0.53 ([11]: fig. 19j).

Character 567: 0.59 ([11]: fig. 18b).

Character 569: 0.54 ([11]: fig. 18b).

Character 571: 0.70 ([11]: fig. 18b).

Character 574: 1.13 ([11]: fig. 18b).

Character 583: 2.56 ([11]: fig. 18b).

Megachirella wachtleri

Character 100: 2.02 ([5]: Extended Data Fig. 1f).

Character 177: 41° ([5]: 3D model).

Character 416: 0.28 ([5]: 3D model).

Character 435: 0.75 ([5]: 3D model).

Character 448: 0.45 ([5]: Extended Data Fig. 2k, l).

Salvator rufescens

Character 2: 0.37 (MACN-Pv Ac 1/0391).

Character 20: 0.40 (MACN-Pv Ac 1/714).

Character 21: 0.42 (MACN-Pv Ac 1/714).

Character 28: 1.73 (MACN-Pv Ac 1/714).

Character 76: 0.69 (MACN-Pv Ac 1/714).

Character 177: ca 70º (MACN-Pv Ac 1/714).

Character 331: 2.50−2.59 (MACN-Pv Ac 1/0391).

Character 351: 2.70−2.87 (MACN-Pv Ac 1/0391).

Character 352: 2.77−3.19 (MACN-Pv Ac 1/0391).

Character 377: 1.18−1.23 (MACN-Pv Ac 1/0391).

Character 379: 1.75−1.78 (MACN-Pv Ac 1/0391).

Character 387: 3.13 (MACN-Pv Ac 1/0391).

Character 410: 0.14 (MACN-Pv Ac 1/0391).

Character 416: 0.30 (MACN-Pv Ac 1/714).

Character 424: 0.26 (MACN-Pv Ac 1/714).

Character 435: 0.74 (MACN-Pv Ac 1/714).

Character 446: 0.51 (MACN-Pv Ac 1/714).

Character 448: 0.28 (MACN-Pv Ac 1/714).

Character 458: 0.17 (MACN-Pv Ac 1/153).

Character 463: 1.19 (MACN-Pv Ac 1/391).

Character 473: 2.58 (MACN-Pv Ac 1/391).

Character 482: 1.96 (MACN-Pv Ac 1/391).

Character 489: 1.16 (MACN-Pv Ac 1/391).

Character 490: 1.22 (MACN-Pv Ac 1/391).

Character 510: 0.24 (MACN-Pv Ac 1/391).

Character 516: 0.78 (MACN-Pv Ac 1/391).

Character 556: 0.49 (MACN-Pv Ac 1/ s/n).

Character 567: 0.70 (MACN-Pv Ac 1/714).

Character 569: 0.60 (MACN-Pv Ac 1/714).

Character 571: 0.72 (MACN-Pv Ac 1/714).

Character 574: 1.11 (MACN-Pv Ac 1/714).

Character 581: 0.74 (MACN-Pv Ac 1/925).

Character 583: 1.69 (MACN-Pv Ac 1/925).

Sphenodon punctatus

Character 2: 0.37 (Morphosource 3D model skeleton).

Character 20: 0.29 (cast).

Character 21: 0.46 (cast).

Character 75: 16 (cast).

Character 76: 0.75 (cast).

Character 100: 3.36 (cast).

Character 177: 75.8º (Morphosource 3D model skull).

Character 266: 0.19 (cast).

Character 331: 0.93–0.94 (Morphosource 3D model skeleton).

Character 351: 1.18–1.29 (Morphosource 3D model skeleton).

Character 352: 1.48–1.58 (Morphosource 3D model skeleton).

Character 377: 0.84–0.94 (Morphosource 3D model skeleton).

Character 379: 1.27–1.31 (Morphosource 3D model skeleton).

Character 387: ca. 4.16 (cast).

Character 410: 0.08 (Morphosource 3D model skeleton).

Character 416: 0.29 (cast).

Character 424: 0.37 (cast).

Character 435: 0.62 (cast).

Character 446: 0.74 (Morphosource 3D model skeleton).

Character 448: 0.37 (Morphosource 3D model skeleton).

Character 458: 0.17 (cast).

Character 463: 0.57 (cast).

Character 472: 2.22 (cast).

Character 482: 1.90 (cast).

Character 489: 1.16 (cast).

Character 490: 1.11 (cast).

Character 510: 0.20 (cast).

Character 516: 0.65 (cast).

Character 567: 0.64 (Morphosource 3D model skeleton).

Character 569: 0.63 (Morphosource 3D model skeleton).

Character 571: 0.81 (Morphosource 3D model skeleton).

Character 574: 1.05 (Morphosource 3D model skeleton).

Character 581: 0.77 (Morphosource 3D model skeleton).

Character 583: 2.40 (Morphosource 3D model skeleton).

Supplementary References

1. Ezcurra, MD. 2016 The phylogenetic relationships of basal archosauromorphs, with an emphasis on the systematics of proterosuchian archosauriforms. *PeerJ* **4**, e1778.
2. Butler RJ, Ezcurra MD, Liu J, Sookias RB, Sullivan C. 2019 The anatomy and phylogenetic position of the erythrosuchid archosauriform *Guchengosuchus shiguaiensis* from the earliest Middle Triassic of China. *PeerJ* **7**, e6435.
3. Pritchard AC, Gauthier JA, Hanson M, Bever GS, Bhullar B-AS. 2018 A tiny Triassic saurian from Connecticut and the early evolution of the diapsid feeding apparatus. *Nat. Commun.* **9**, 1213.
4. Estes R, De Queiroz K, Gauthier J. 1988 Phylogenetic relationships within Squamata. In *Phylogenetic Relationships of the Lizard Families* (eds R Estes, G Pregill), pp. 119−281. Stanford: Stanford University Press.
5. Simões TR, Caldwell MW, Tałanda M, Bernardi M, Palci A, Vernygora O, Bernardini F, Mancini L, Nydam RL. 2018 The origin of squamates revealed by a Middle Triassic lizard from the Italian Alps. *Nature* **557**, 706−709.
6. Pritchard AC, Turner AH, Nesbitt SJ, Irmis RB, Smith ND. 2015 Late Triassic tanystropheids (Reptilia, Archosauromorpha) from northern New Mexico (Petrified Forest Member, Chinle Formation) and the biogeography, functional

morphology, and evolution of Tanystropheidae. *J. Vertebr. Paleontol.* **35**, e911186.

7. Gauthier JA, Kearney M, Maisano JA, Rieppel O, Behlke AD. 2012 Assembling the squamate tree of life: perspectives from the phenotype and the fossil record. *Bull., Peabody Mus. Nat. Hist.* **53**, 3−309.
8. Benton MJ. 1985 Classification and phylogeny of the diapsid reptiles. *Zool. J. Linn. Soc.* **84**, 97−164.
9. Gauthier JA, Estes R, De Queiroz, K. 1988 A phylogenetic analysis of Lepidosauromorpha. In *Phylogenetic Relationships of the Lizard Families* (eds R Estes, G Pregill), pp. 119−281. Stanford: Stanford University Press.
10. Fraser NC. 1988 The osteology and relationships of *Clevosaurus* (Reptilia: Sphenodontida). *Phil. Trans. R. Soc. Lond. B* **321**, 125−178.
11. O'Brien A, Whiteside DI, Marshall JE. 2018 Anatomical study of two previously undescribed specimens of *Clevosaurus hudsoni* (Lepidosauria: Rhynchocephalia) from Cromhall Quarry, UK, aided by computed tomography, yields additional information on the skeleton and hitherto undescribed bones. *Zool. J. Linn. Soc.* **183**, 163−195.
