# Supplementary figures and images for: Colobops: a juvenile rhynchocephalian reptile (Lepidosauromorpha), not a diminutive archosauromorph with an unusually strong bite
Source: R Soc Open Sci. 2020 Mar 25;7(3):192179. doi: 10.1098/rsos.192179 (PMC7137947; doi:10.1098/rsos.192179)

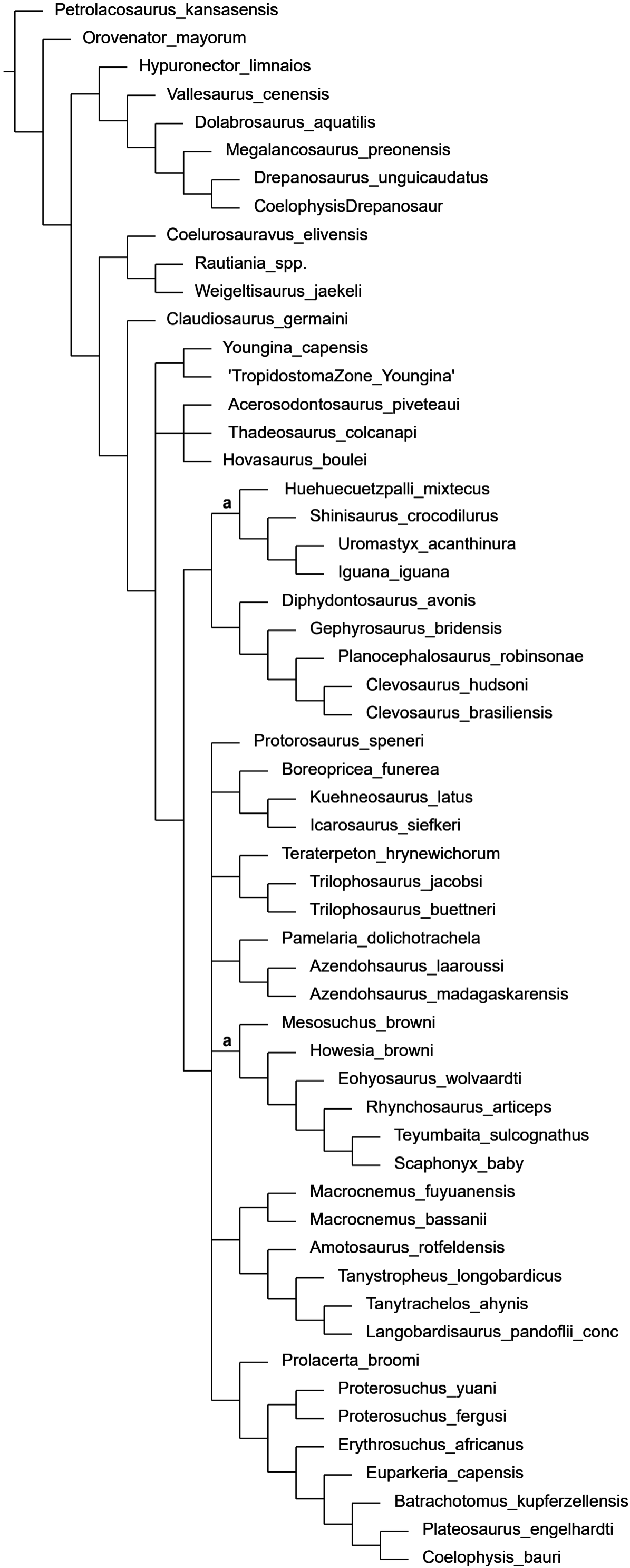

**a: alternative placements of Colobops\_noviportensis**

Supplement: Supplement Figure S2 [file rsos192179supp2.pdf]

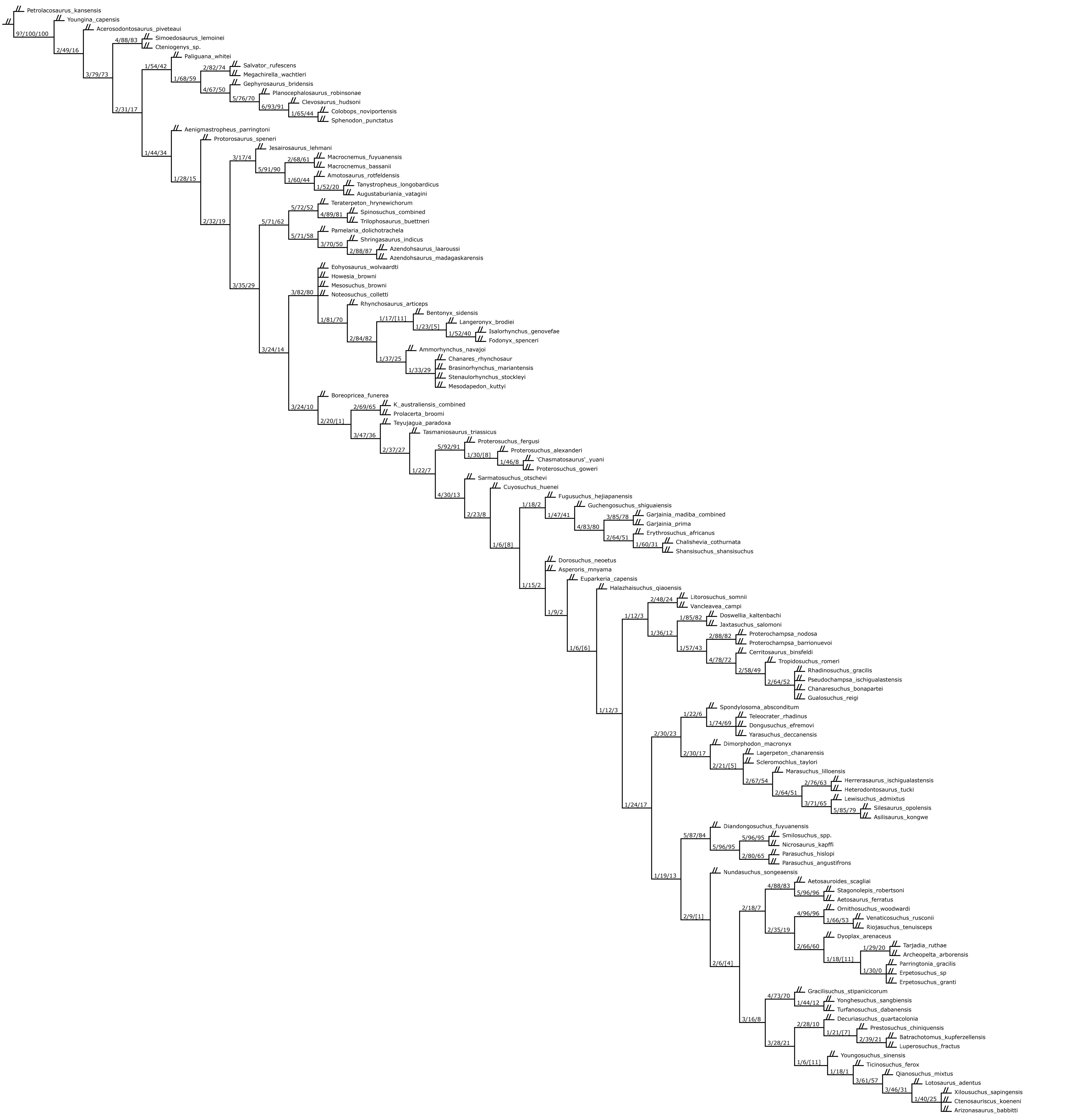

Supplement: Supplement Figure S3 [file rsos192179supp3.pdf]

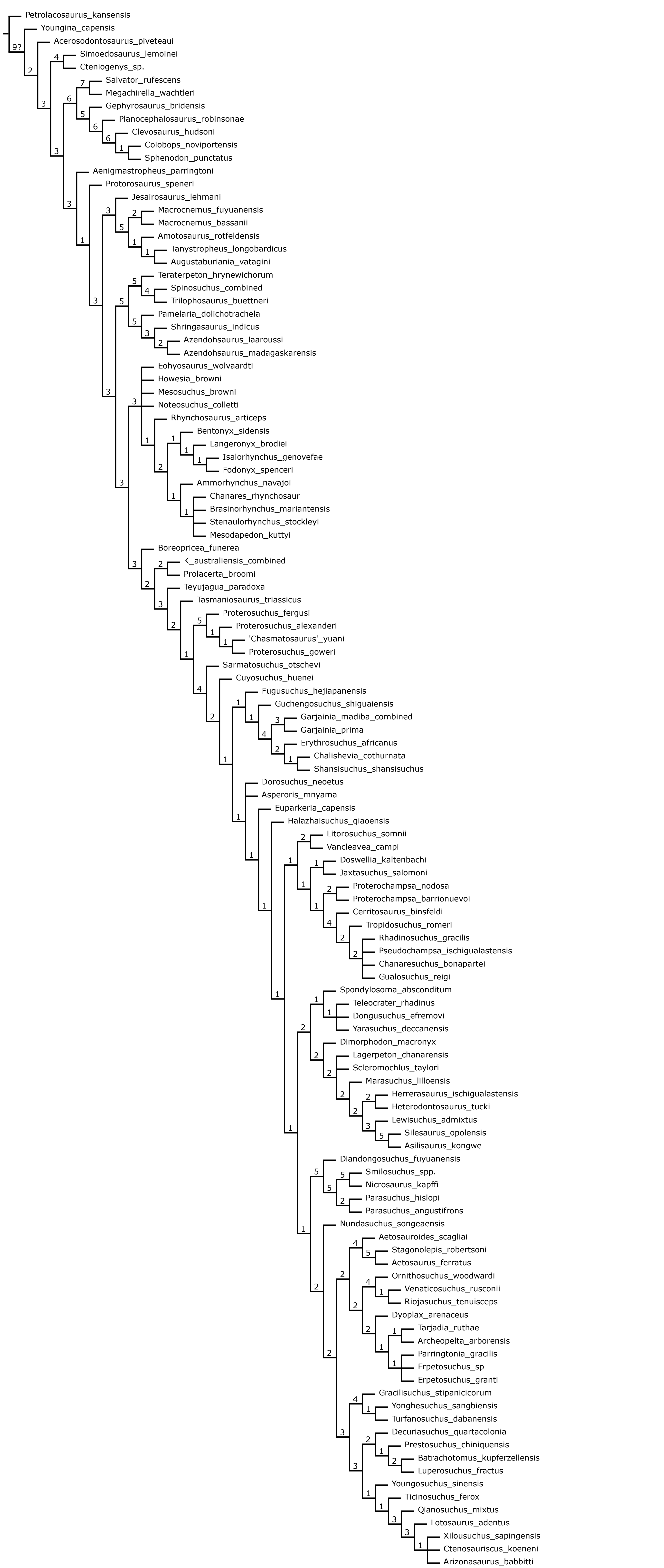

Supplement: Supplement Figure S4 [file rsos192179supp4.pdf]
